# Management of tympanic membrane retractions: a systematic review

**DOI:** 10.1007/s00405-021-06719-3

**Published:** 2021-03-10

**Authors:** Ahmed B. Bayoumy, Christianne C. A. F. M. Veugen, Erwin L. van der Veen, Jan-Willem M. Bok, Jacob A. de Ru, Hans G. X. M. Thomeer

**Affiliations:** 1 Department of Otolaryngology, Ministry of Defense, Central Military Hospital, Utrecht, The Netherlands; 2 Department of Otolaryngology-Head and Neck Surgery, University Medical Centre Utrecht, Utrecht, The Netherlands; 3 Department of Otolaryngology, Flevoziekenhuis, Almere, The Netherlands; 4 UMC Utrecht Brain Center, Utrecht University, Utrecht, The Netherlands

**Keywords:** Tympanic membrane retractions, Retraction pockets, Tympanoplasty, Watchful, Waiting, Ventilation tubes

## Abstract

**Importance:**

Tympanic membrane retraction (TMR) is a relatively common otological finding. However, no consensus on its management exists. We are looking especially for a treatment strategy in the military population who are unable to attend frequent follow-up visits, and who experience relatively more barotrauma at great heights and depths and easily suffer from otitis externa from less hygienic circumstances.

**Objective:**

To assess and summarize the available evidence for the effectiveness of surgical interventions and watchful waiting policy in patients with a tympanic membrane retraction.

**Evidence review:**

The protocol for this systematic review was published at Prospero (207859). PubMed, Embase, and the Cochrane Database of Systematic Reviews were systematically searched from inception up to September 2020 for published and unpublished studies. We included randomized trials and observational studies that investigated surgical interventions (tympanoplasty, ventilation tube insertion) and wait-and-see policy. The primary outcomes of this study were clinical remission of the tympanic membrane retraction, tympanic membrane perforations and cholesteatoma development.

**Findings:**

In total, 27 studies were included, consisting of 1566 patients with TMRs. We included data from 2 randomized controlled trials (76 patients) and 25 observational studies (1490 patients). Seven studies (329 patients) investigated excision of the TMR with and without ventilation tube placement, 3 studies (207 patients) investigated the wait-and-see policy and 17 studies (1030 patients) investigated tympanoplasty for the treatment of TMRs.

**Conclusions and relevance:**

This study provides all the studies that have been published on the surgical management and wait-and-policy for tympanic membrane retractions. No high level of evidence comparative studies has been performed. The evidence for the management of tympanic membrane retractions is heterogenous and depends on many factors such as the patient population, location and severity of the TMR and presence of other ear pathologies (e.g., perforation, risk of cholesteatoma and serous otitis media).

**Supplementary Information:**

The online version contains supplementary material available at 10.1007/s00405-021-06719-3.

## Background

An 18-year-old cadet from the Navy presents at the military otolaryngology clinic with minimal hearing loss, objectified during the military entrance medical test. During clinical examination a tympanic membrane retraction is seen. Since these are mild abnormal findings, the question is whether a watchful waiting strategy is justified or that a tympanoplasty should be advised to create a safe ear with good hearing for the future. This case shows an example of a frequently encountered otological finding that might cause debate regarding treatment necessity in, but not confined to, the TMR.

The tympanic membrane consists of three layers: the epidermal layer, the mucosal layer and the lamina propria. In both the pars tensa and pars flaccida, the epidermal layers are similar. These epidermal cells have a specialized function; they have the potential for lateral migration. The cells migrate outwards from the center to the cartilaginous portion of the external ear. This migration process is responsible for the regenerative and self-cleaning abilities of the tympanic membrane. The mucosal layer is an extension of the middle-ear mucosal lining, and consists of layers of epithelial cells [1]. The biological difference between the pars tensa and pars flaccida can be found in the lamina propria. In the pars tensa, the lamina propria varies in thickness depending on the anatomic area. In the posterosuperior quadrant the thickness is approximately 40 µm, while near the annulus the thickness is 90 µm. The fibrous layer of the pars tensa is attached to handle of the malleus [2]. Retractions in pars tensa can lead to clinical symptoms such as hearing loss, especially with promontory contact which is a reason to refuse military duty (e.g., pilots and divers).

TMRs are also frequently observed in the pars flaccida. The lamina propria of the pars flaccida consists primarily of loose and unorganized connective tissue and is thicker than the pars tensa. The unorganized fibers in its connective tissue makes the structural arrangements within the pars flaccida quite weak and more likely to retract [1]. Furthermore, the absence of a surrounding tympanic annulus also contributes to this weakness. However, retractions in the pars flaccida are usually asymptomatic and are therefore underreported. In the literature, prevalence rates of TMRs in the civilian population have been described for pars tensa and pars flaccida retractions, respectively of 0.3–3.7% and 14–26% [3]. The prevalence of TMRs in the military is not described in literature. In the majority of cases TMRs remain stable, and spontaneous recovery occurs in approximately 30% of mild TMR cases (Sade grade I) [4–6]. However, if TMRs are more severe, it can lead to conductive hearing loss, tympanic membrane perforations, erosion of the ossicular chain, higher rate of external otitis due to failure of the normal ‘cleaning’ of the tympanic membrane outer edges and formation of cholesteatoma. TMRs are of substantial clinical importance in the formation of cholesteatoma; about 4% of TMRs progress into cholesteatoma [7]. Considering the potential progression to cholesteatoma, correct diagnosis and proper management of TMRs is crucial. However, there is no consensus among ENT-physicians regarding the best strategy for treating TMRs [8]. Some advocate surgical intervention to prevent progression of the retraction, others a watchful waiting policy avoiding surgery as long as possible. In the military population, due to often changing personal schedules because of deployment and placement issues, routine follow-up visits can be difficult to adhere to. Military patients also experience relatively more barotrauma due to blast injuries and exposure to great heights and depths, and suffer easily from otitis externa from less hygienic circumstances [9–12]. For some of the military personnel, it is not possible to receive a ventilation tube because an intact tympanic membrane is required for their duties (e.g., divers in the Navy). Hence, there is a need for evidence that supports either interventions or wait-and-see policy for the (military) patient to make appropriate clinical decisions. The aim of this systematic review is to investigate the effectiveness of surgical interventions in patients with a tympanic membrane retraction and to assess the evidence of the wait-and-see policy.

## Methods

### Data sources and searches

We systematically searched PubMed and Embase, from inception up to September 2020 for investigating (non)-surgical interventions for TMRs. This electronic search strategy was augmented by a manual examination of references cited in articles, recent reviews, editorials, and meta-analyses. No restrictions were imposed on the language, study period, or sample size.

### Study selection and outcome definition

Two investigators (AB and CV) independently screened titles and abstracts, identified duplicates, reviewed full articles, and determined their eligibility. Any discrepancies were resolved by reaching a consensus regarding the inclusion or exclusion of a trial. The last search was performed in September 2020. An algorithm for inclusion and exclusion criteria was used according to the PICO-principle:

- Population: patients of all ages treated primarily for TMRs. Studies that reported other indications (e.g., cholesteatoma, perforation or serous otitis media) were included only if the treatment of TMRs was analyzed separately.
- Intervention: surgical intervention for TMRs or wait-and-see policy.
- Comparator: (non)-surgical intervention (including tympanoplasty, ventilation tubes and wait-and-see policy).
- Outcome: the primary outcome was complete remission of the TMR, and secondary outcomes were hearing recovery, progression to cholesteatoma, progression to perforation, external otitis or recurrence of TMR.
- Study design: comparative and observational studies were included in this review. Case reports or (narrative) reviews were excluded.

### Data extraction and quality assessment

The data were compiled using a standardized form to extract the following study characteristics: study design, number of patients, treatment, graft material, age category, outcome definitions and patients’ demographics. The quality of the eligible studies was assessed using the Cochrane Collaboration’s tool for assessing risk of bias for RCTs (Supplement 1). The ROBINS-I tool [13] and the Strengthening the Reporting of Observational Studies in Epidemiology (STROBE) [14] checklist were used to assess the quality of nonrandomized studies. The risk of bias was assessed in six main areas: sequence generation, allocation concealment, blinding of participants, personnel and outcome assessors, incomplete outcome data and selective outcome reporting. This study was reported according to the PRISMA statement [15].

## Results

### Search findings

**Fig. 1:**
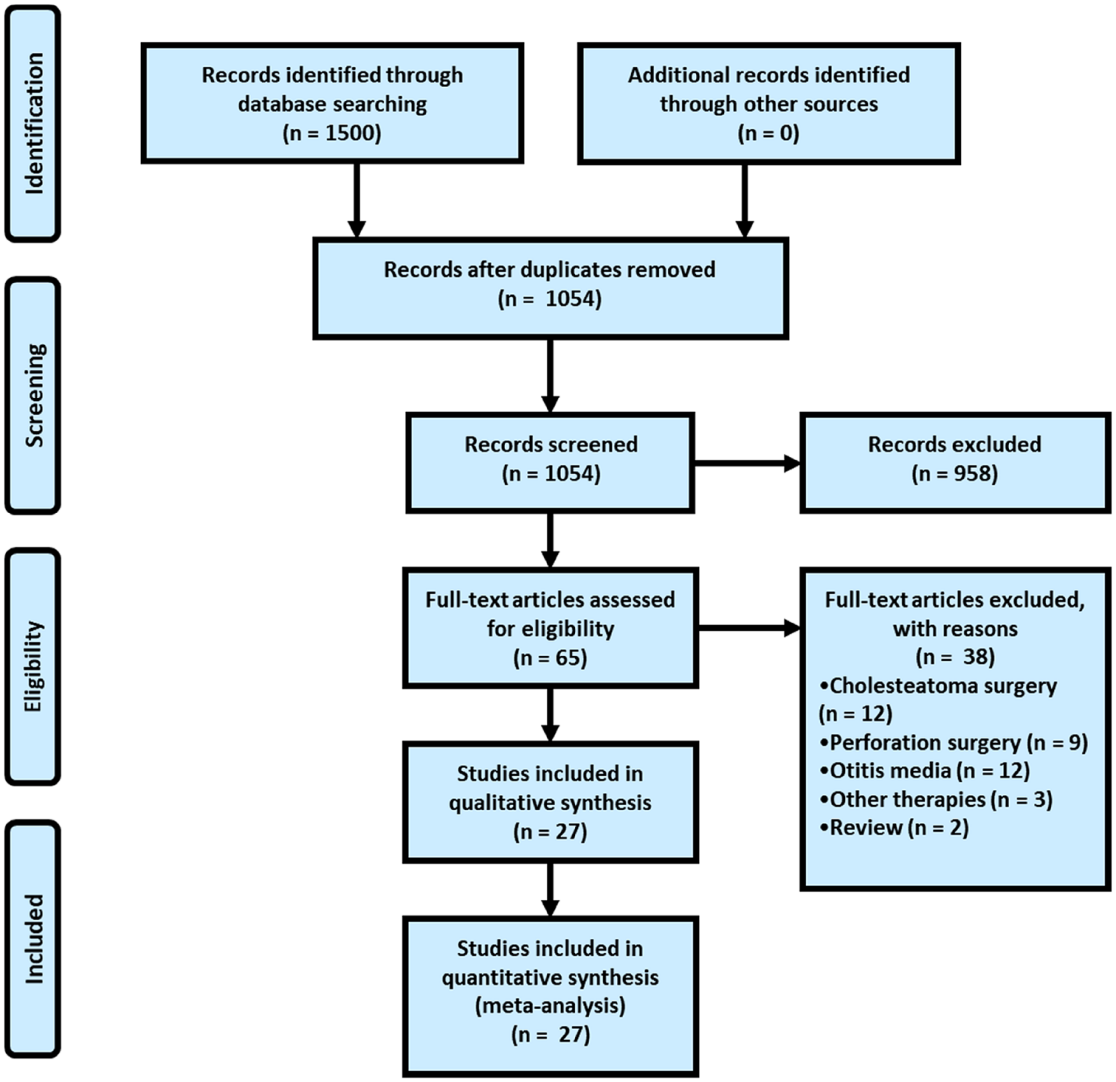
PRISMA flowchart of screened and included studies [13]

A total of 1054 citations were identified. Among these, 65 articles were considered for full review, of which 27 met the inclusion criteria. In total, 27 studies were included, consisting of 1566 patients with TMRs. The flowchart of this study can be found in Fig. 1. The main reasons for exclusion were studies that performed surgeries for perforations, cholesteatoma or serous otitis media (tube insertion).

### Study characteristics and risk of bias assessment

**Fig. 2:**
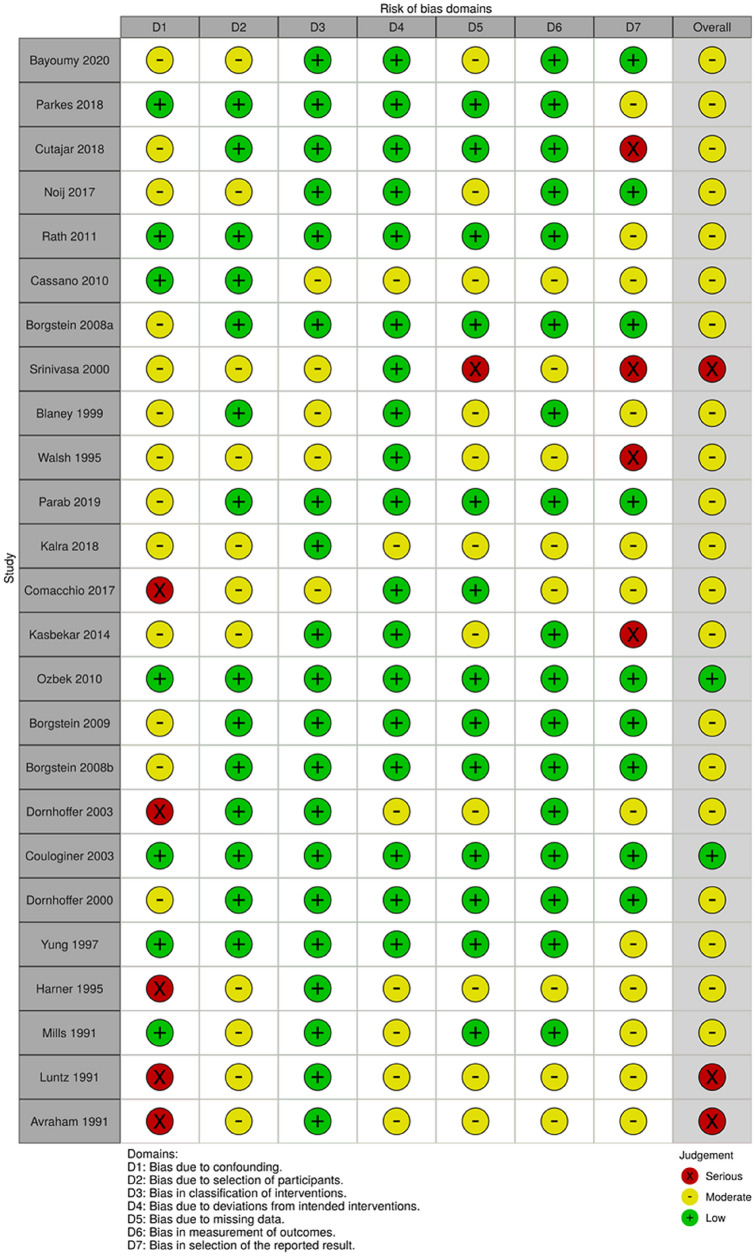
Risk of bias assessment of non-randomized studies (*n* = 27, 1566 patients) in concordance with the ROBIN-I risk of bias tool [11]

The main characteristics of the individual studies are summarized in Table 1. Two of the non-randomized studies had low risk of bias, 20 studies had moderate risk of bias and 3 studies had high risk of bias (Fig. 2). The two RCTs were classified as having high risk of bias (Supplement 4).

**Table 1:** Studies with tympanoplasty (*n* = 17, 1030 patients)

Author	Age	Sex	Location TMR	Intervention	Comparator	Follow-up	Remission TMR (%)	Audiometry (dB, ABG)	Other remarks
Barbara [14] (*n* = 30)	29–63	M: 13 F: 12	PF	TP	WS	12 months	TP: 100% WS: 3 TMRs (33%) worsened	N.R.	Hearing assessment showed absence of deterioration in TP
Elsheikh [15] (*n* = 46)	TP + VT 27 (9.3) TP: 29 (7.8)	M: 29 F: 17	PT/PF	TP (PCG) + VT	TP	1 months	100%	TP Pre-op: 22.7 dB Post-op: 10.9 dB TP + VT Pre-op: 24.6 dB Post-op:12.2 dB	Conductive hearing los TP + VT: 2 (9%) TP: 3 (13%)
Parab [28] (*n* = 41)	32,6 (4,5)	M: 23 F: 18	PT/PF	Endoscopic TP tragal PCG	N.A.	3 years	100%	Pre-op 24.5 dB (4.3) Post-op 14.1 dB (5.9) Gain: 10.4 dB	
Kalra [39] (*n* = 20)	10–40	M: 12 F: 8	PT/PF	TP PCG	N.A.	3 months	Recurrence in 6 (30%) Persistent perforation in 2 (10%)	Hearing was improved up to 15 dB in 16 (80%)	
Comacchio [40] (*n* = 25)	47.6 (19.1)	M: 17 F: 7	PT	TP	N.A.	6 years	100%	N.R.	
Kasbekar [41] (*n*= 42)	38 (8–66)	N.R.	PT/PF	TP PCG	N.A.	38 months	Recurrence TMR: 1 (2%)	Pre-op: 24 dB Post-op: 17.3 dB Gain: 6.7 dB	Progression to CST: 1 (2%)
Özbek [16] (*n* = 56)	23.8 (10.8)	M: 24 F: 30	PT	TP (type I, II, III) tragal PCG	N.A.	44.5 months	Healed created perforations in 51 (91%)	Pre-op: 28.4 dB Post-op: 16.9 dB Gain: 11.5 dB	
Borgstein [20] (*n* = 169)	9.6 (3.4)	M: 57 F: 70	PT	TP (tragal PCG or TF)	N.A.	7–15 months	N.A.	Audiometry per disease stage provided in study	Stage I: progression in 10 (22%), CST 2 (4%) Stage II: progression in 3 (19%) Stage III: CST 4 (13%) Stage IV: CST 25 (24%)
Borgstein [42] (*n* = 46)	10.4 (3.4)	M: 16 F: 26	PT	TP	N.A.	6 months	N.A.	Non-erosion group Pre-op: 10.0 dB (SD 9.8) Postop: 5.9 dB (SD 8.3) Incus erosion group Preop: 20.1 dB (SD 13.3) Postop: 13.8 dB (SD 9.1)	
Dornhoffer [43] (*n* = 98)	N.R.	N.R.	PT	TP	N.A.	15 months	N.R.	Preop: 20.2 (SD 10.9) Postop: 14.2 (SD 10.2) *P* < 0.05	Perforation: 1 (1%) Postop tube insertion: 7 (7%) Intra-op tube insertion: 12 (12%)
Couloginer [21] (*n* = 60)	10 (3.5)	NR	PT/PF	TP (tragal or conchal PCG)	N.A.	27 months (18)	Recurrence: 7 (12%)	Preop: 26 dB (SD 12) Postop: 22 dB (SD 12) Gain: 4 dB	Revision surgery for poor hearing outcome in 4 (7%)
Dornhoffer [17] (*n* = 63)	24 (5–78)	M: 38 F: 27	PT	Type I TP	TP + ossicular reconstruction	26 months (12–48)	5 TMRs persisted	Preop: 20.6 dB (SD 11.3) Postop: 10.7 dB (SD 5.6) Gain: 9.9 dB Gain I: 6.5 dB Gain C: 15.3 dB	12 ears (19%) required VT
Yung [18] (*n* = 72)	N.R.	N.R.	PT	TP (type III/IV/PCG)	N.A.	3–8 years	Posterior TMR: 26 (81%) Complete atelectatic TM: 13 (33%)	Posterior TMR Preop: 26 dB Postop: 10 dB Complete atelectatic TM: Preop: 34 dB Postop: 24 dB	Posterior TMR: cholesteatoma: 1 (3%) Recurrent TMR: 1 (3%) Complete atelectatic TM: Recurrent TMR: 20 (50%) cholesteatoma in 2 (5%)
Harner [44] (*n* = 22)	20 (6–60)	M: 13 F: 9	PT	TP (tragal PCG)	N.A.	N.R.	N.R.	Preop: 19 (range 6–33) Postop: 14 (range 0–33)	
Mills [45] (*n* = 93)	39 (6–88)	M: 27 F: 50	PT	TP (*n* = 7) PCG	WS	12 months	TP: TMR grade reduced in 4, stable TMR in 2 and complete remission in 2	N.R.	
Luntz [46] (*n* = 36)	4–13	N.R.	PT	TP	N.A.	50–55 months	Remission: 51 (61%) Deteriorated: 5 (6%)	N.R.	
Avraham [47] (*n* = 111)	N.R.	N.R.	PT	TP + mastoidectomy [MD] (*n* = 27)	TP (*n* = 84)	53.1 months	TP + MD Normal TM: 6 (22%) TP: Normally TM: 51 ears (61%)	N.R.	

*TP* tympanoplasty, *WS* wait-and-see, *PT* pars tensa, *PF* pars flaccida, *CST* cholesteatoma, *PCG* perichondrium cartilage graft, *TF* temporalis fascia, *VT* ventilation tubes

### Tympanoplasty

This intervention involves the excision of the TMR, and reconstruction of the tympanic membrane with a cartilage graft. Seventeen studies (*n* = 1030) were published that performed tympanoplasties for TMRs, of which two were RCTs (*n* = 76 ears). The first RCT by Barbara et al. [16] consisted of 30 patients, aged between 29 and 63 years old. One group of 15 patients underwent lateral attic reconstruction surgery with tragal cartilage and the other group underwent a wait-and-see policy. The duration of follow-up was 12 months in this study. All patients in the surgical group had preoperative hearing levels within the normal range. No patients showed worsening of hearing postoperatively. Only one patient suffered from a postoperative complication with a purulent discharge. Five patient received revision surgery due to hypodense abnormalities found on CT. No pathological tissue was found behind in the cartilage in all five patients. During in the entire follow-up, two patients showed widening of the bony epitympanic erosion and one patient showed skin ingrowth. These patients were planned for surgery. No deterioration of hearing loss was found in the wait-and-see group.

The second prospective RCT performed by Elsheikh et al. [17] compared perichondrium/cartilage tympanoplasty plus ventilation tube (*n* = 23) versus perichondrium/cartilage tympanoplasty (*n* = 23). The mean pre- and postoperative hearing air–bone gap was 24.6 dB (SD 9.7) and 12.2 dB (SD 7.0) in the tympanoplasty and ventilation tube group. The mean gain in air–bone gap was statistically significant (+ 12.4 dB, *P* < 0.05). In the tympanoplasty group, the mean pre- and postoperative hearing air–bone gap was 24.6 dB (SD 9.7) and 12.2 dB (SD 7.0). The mean gain in air–bone gap was also statistically significant in this group (+ 11.8 dB, *P* < 0.05). However, no difference was found between the two groups. They did not observe any postoperative graft failure or recurrence of the TMR. Four studies [18–21] used different tympanoplasty techniques and two studies [22, 23] used different grafting materials.

### Excision of TMR with ventilation tube insertion

This technique involves an excision of the TMR, after which a ventilation tube is placed. The rationale behind this technique is that it may recover the difference between atmospheric pressure and middle-ear pressure, and thus abolish the driving force causing the tympanic membrane to be retracted. Six studies [24–29] have been described that excised the TMR and subsequently placed ventilation tubes. Rath et al. [25] was the only prospective study in 40 children, of whom 38 (95%) of the created perforation healed. There were six recurrences (15%) of TMRs, which all required revision interventions. Four of those newly created perforations healed. The pre-operative air–bone gap was 22.4 dB, while the post-operative air–bone gap was 9.7 dB (hearing gain 12.7 dB). No cholesteatoma developed during this study. In all six studies mentioned above (*n* = 243), it can be summarized that the created perforations remain persistent in 3–13% and that recurrences of the TMR occur in 11–25% of operated patients. The summary can be found in Table 2 [24–29].

**Table 2:** Studies with excision of the TMR with(out) ventilation tube insertion (*n* = 7, 329 patients)

Author	Age	Sex	Location TMR	Comparator	Follow-up	Remission TMR (%)	Audiometry (dB, ABG)	Other remarks
Noij [22] (*n* = 77)	48 (18–71)	M: 31 F: 31	PT	N.A.	1 year	Healed created perforation in 72 (94%)	Pre-op: 13.1 Post-op: 11.5 Gain: 1.6	5 persistent perforation, three required myringoplasty
Rath [23] (*n* = 40)	7.2 (3–14)	M: 14 F: 16	PT	N.A.	16.1 months (6–29)	6 recurrences (15%), 6 s interventions (15%), 4 of 6 healed	Pre-op: 22.4 dB Post-op: 9.7 dB Gain: 12.7 dB	38/40 created perforation closed 0% cholesteatoma
Cassano [25] (*n* = 45)	5–12	M: 19 F: 18	PT	WS	24 months	VT: 15 (94%) WS: 14 (35%)	N.A	Recurrence in 5 ears (11%)
Borgstein [39] (*n* = 86)	9 (4–18)	M: 28 F: 34	PT	N.A	7–15 months	94% of created perforations healed Recurrence in 17 (20%)	Significant improvement of ABG at final follow-up (*P* < 0.01)	Postoperative discharge in the operated ear: 8.1%
Srinivasan [24] (*n* = 31)	9 (3–14)	M: 16 F: 10	PT	N.A.	16 months (range: 8–34)	Recurrence in seven ears, one ear with persistent perforation. Recurrence free rate of 74%	N.R.	
Blaney [26] (*n* = 39)	7 (3–13)	M: 17 F: 14	PT	N.A.	27 months (1–52)	34 of 39 created perforations healed (87%) 13 recurrence TMRs, 4 underwent revision surgery	Pre-op > 30 dB loss: 16 (49%) Post-op > 30 dB loss: 7 (21%) Pre-op 0–10 dB loss: 1 (3%) Post-op 0–10 dB loss: 5 (15%)	
Walsh [27] (*n* = 11)	6.6 (4–11)	M: 5 F: 4	PT	N.A	16 months (10–24)	Completely healed: 10 Recurrence in two ears (2%)	Hearing improvement in 8 ears (mean improvement in air-conduction 16 dB)	

*PT* pars tensa, *PF* pars flaccida, *CST* cholesteatoma, *PCG* perichondrium cartilage graft, *TF* temporalis fascia, *VT* ventilation tubes, *WS* wait-and-see policy

### Endoscopic tympanoplasty

Parab and Khan [30] performed a prospective study in 41 ears that underwent endoscopic tympanoplasty with tragal perichondral graft. The mean age of patients was 32.6 (range 14–47) and 24 ears had erosion of the ossicular chain. Twenty-eight TMRs were located in pars tensa and 13 TMRs were located in pars flaccida. The distribution of the TMRs according to Sade were grade II in 4 patients; grade III in 23 and grade IV in 14 patients. After a follow-up period of 3 years, all the TMRs went in remission. The pre-operative air–bone gap was 24.5 dB, while the post-operative air–bone gap was 14.1 dB (hearing gain 10.4 dB).

### Wait-and-see policy

The wait-and-see policy is a conservative management policy in which patients with TMRs are followed-up at the otolaryngology clinic without invasive surgery or ventilation tube placement. The wait-and-see policy was used in three studies (Table 3) [5, 6, 31] (*n* = 207). In 76–96% of TMRs remain stable or improved during follow-up, complete remission rates vary between 0 and 38% [5, 6, 31]. One study found that no adult ears had complete remission, while 31% of children’s ears did improve [31]. Improvement of audiometry is related to the grading of the TMR. The perforation rate was found to lie between 4–6%. Progression to cholesteatoma occurred in 1–5% of patients. In these TMRs that developed into cholesteatoma had contact with the promontory [5, 6]. Parkes et al. [5] did not find any relationship between hearing outcomes and the condition (Sade grade I versus Sade grade > I) of the TMR (*P* = 0.60). Bayoumy et al. [6] reported that in 81 TMRs the initial mean air–bone gap was 17.9 dB, which improved to 15.5 dB at final follow-up (*P* = 0.08). A significant difference was found in hearing improvement between patients presenting with Sade grade I versus Sade grade III + IV (8.3 versus − 2.4 dB, *P* = 0.001). Furthermore, children (≤ 18 years) had higher hearing gains at final follow-up compared to adults (8.0 versus − 1.3 dB, *P* = 0.0001).

**Table 3:** Studies with wait-and-see policy (*n* = 3, 207 patients)

Author	Age	Sex	Location TMR	Follow-up	Remission TMR (%)	Audiometry (dB, ABG)	Other remarks
Bayoumy [4] (*n* = 81)	23 (14–47)	M: 42 F: 39	PT/PF	64 months	96% with improved or stable TMRs 10% complete remission	First-visit: 17.9 dB Last visit: 15.5 dB Audiometry by Sade grade was provided in the study	Progression to CST in 1 (1%) Progression to perforation in 5 (6%)
Parkes [3] (*n* = 37)	15 (9–21)	N.R.	PT	6.4 years (0.75–7.6)	76% with improved or stable TMRs	Initial PTA stable/improved: 11.2 dB	Progression to CST in 2 (5%)
Cutajar [29] (*n* = 89)	12.6	N.R.	PT	10 years	No adult ear in remission 20 children ears improved (31%)	N.R.	The proportion of children whose ears improved at 5 years was 0.19 (CI 0.09–0.29), which increased to 0.56 (CI 0.38–0.74) by 10 years

*PT* pars tensa, *PF* pars flaccida, *CST* cholesteatoma

## Discussion

### Review of evidence

In this systematic review, 27 studies were identified with a total of 1530 patients. The data consisted of two randomized controlled trials (76 patients) and 25 observational studies (1490 patients). Seven studies (329 patients) investigated excision of the TMR with(out) ventilation tube placement, 3 studies (207 patients) investigated the wait-and-see policy and 17 studies (1030 patients) investigated tympanoplasty for the treatment of TMRs.

It has been 10 years ago since the last Cochrane Systematic Review [8] regarding this subject was published. Another systematic review was published two years later by Neumann and Yung [32]. These two systematic reviews only included two RCTs [16, 17] and excluded all the non-randomized comparative and observational studies. Whereas, our systematic review included all comparative and non-comparative studies with a (non)surgical intervention for TMRs.

In total, seven studies (*n* = 329) were included that had investigated excision of the TMR with ventilation tube insertion. For these studies, it could be summarized that the created perforations remain persistent in 3–13% and that recurrences of the TMR occur in 11–25% of operated patients. These results show that approximately 3–25% of patients treated with this technique might need a re-intervention for either the persistent perforation or the recurrence of the TMR. This outcome may be important to consider during shared decision-making. For military personnel, this could mean additional delay and reduction of the employability.

The wait-and-see policy was used in three studies (*n* = 207). In 76–96% of TMRs remain stable or improved during follow-up, complete remission rates varied between 0 and 38%.

Improvement of audiometry is related to the grading of the TMR. It was found that in patients with mild retraction pockets (Sade grade I), hearing improved over time and that in a small portion of these patients the retraction completely resolved spontaneously [6]. Long-term results indicate that the wait-and-see policy differs in outcomes depending on the severity of the TMRs. Cholesteatoma developed occurs in approximately 1–5% of patients. In both studies published by Parkes et al*.* [5] and Bayoumy et al. [6], the TMRs that developed into cholesteatoma had promontory contact. It seems that these retractions might have higher risk for developing into cholesteatoma. However, larger observational studies are needed to confirm these results.

In mild retractions (Sade grade I) of the posterosuperior quadrant of pars tensa, acceptable outcomes were found that might justify the use of the wait-and-see policy in this group of patients. Military patients with a mild TMR can be employed as the risks for otoscopic progression and perforation are relatively low. However, for certain military subpopulations such as divers and pilots, more caution might be taken because these patients are exposed to higher pressure changes on the tympanic membrane. The follow-up of patients can occur after employment, in which the stage of the TMR can be reassessed. If the TMR has progressed, an operation of the TMR may be warranted.

For tympanoplasty, two RCTs have been performed by Barbara et al. [16] and Elsheikh et al. [17].

The outcomes of tympanoplasty with a tragal cartilage reconstruction of the lateral attic wall by Barbara et al. [16] might indicate some reduction of the progression rate of TMRs. However, the results were not statistically significant, and the number of cases was relatively small. Based on this RCT, the tympanoplasty was not significantly better compared to the wait-and-see policy. Elsheikh et al. [17] did not find additional benefits of ventilation tube insertion combined with cartilage tympanoplasty compared to cartilage tympanoplasty alone. These results cannot be generalized to ventilation tubes alone as all patient received cartilage tympanoplasty. For the military patient with a severe TMR, tympanoplasty may still be an option because of the risk of progression of hearing loss, perforations and development of cholesteatoma [5].

### Future directions and research

Parab and Khan [30] published a promising study of endoscopic tympanoplasty for TMRs. They showed that a two-handed endoscopic tympanoplasty can be safely used and good results can be achieved. The two-handed technique can be performed using endoscopic holders which allows the surgeon to use the suction in the left hand, while holding the micro-ear instruments in the right hand. The advantages of the two-handed endoscopic technique is continuous suction and intermittent irrigation during surgery and better visualization of the sinus tympani, facial recess, anterior tympanic cavity and hypotympanum. However, disadvantages include inferior visualization due to a narrow canal and costs of the endoscope holder. Endoscopic tympanoplasty for TMRs is a promising surgical technique, but further studies are needed to confirm these results. One of the causes of TMRs is the eustachian tube dysfunction [33, 34]. Therefore, another promising treatment option that may be combined with the wait-and-see policy might be eustachian tube dilatation. This technique comprises the inflation of a balloon in the cartilaginous part of the Eustachian tube to cause local dilatation. A systematic review by Huisman et al*.* found that this procedure reduces the amount of symptoms and that eustachian tube dysfunction scores decreased in all included studies [35]. Eustachian tube dilatation can be interesting for military patient populations because for this specific patient group a ventilation tube is not an option. However, it still remains unclear whether eustachian tube dilatation improves the tympanic membrane retraction. Huhnd et al. [36] found that in only 31% of the TMRs improved despite self-assessed clinical improvements in 54% of patients who underwent eustachian tube dilatation.

It is evident from the results of this systematic review that more research is needed for the optimal management of TMRs, especially for the military population. TMRs are a common problem in otolaryngology and its surgical management strategies are considerably variable. Numerous issues must be addressed by future research to optimize these surgical strategies. The methodology of studies must be improved using randomized controlled trials which may remove important sources of bias. These surgical interventions should be compared to the wait-and-see policy, especially for mild retractions for which the latter can be ethically and medically justified. In the future, telemedicine can also be implemented in addition to regular follow-up for the monitoring of TMRs in the wait-and see policy [37].

Furthermore, it is important that the study population and retractions are homogenous and clearly defined. The TMRs should be of similar severity and location within in the tympanic membrane. The staging system of TMRs should be comparable and reliable, this is important to evaluate results between different studies. Various useful staging systems have been proposed for TMRs in pars tensa and pars flaccida [38]. The staging systems include the Sade, Charachon, Tos, Dornhoffer and Erasmus classification. These staging systems have been reviewed by Alzahrani et al. [38], we would like to refer to their article for more details about the classification systems. A cross-sectional study performed by James et al. [39] found varying levels of inter- and intra-observer agreement between repeated assessments of tympanic membrane retractions in children with cleft palate for different staging systems.

### Pathophysiology of tympanic membrane retractions: pressure regulation and gas-exchange

The primary function of the tympanic membrane is that it transmits incoming soundwaves from the external ear through the ossicles towards the oval window and into the cochlea. In the cochlea, the soundwaves are transformed into neurological stimuli for the auditory cortex. However, for optimal tympanic membrane function, it is critical that the pressure in the middle ear is maintained at atmospheric level. The middle-ear is a relatively fixed-volume, temperature-stable, biological gas pocket, its pressure is proportional to the contained gas moles [40]. There are four compartments which exchange gasses with the middle ear: the inner ear via the round window, the atmospheric environment through the tympanic membrane, gas-exchange via the mastoid mucosa, and the Eustachian tube (ET) [41–43]. The middle-ear is ventilated by transmucosal gas exchange, a passive, partial-pressure gradient-drive diffusive exchange of gases through the middle-ear mastoid mucosa. The gas-exchange through the middle-ear mastoid mucosa is a relative slow process, which cannot manage abrupt changes in atmospheric pressure [44]. The ET is a 3.5 cm long tube that connects the middle ear cavity with the nasopharynx. The shape of the ET bears a resemblance to an hourglass. It consists out of three components: the bony part close to the middle ear; the fibro-cartilaginous part near the nasopharynx which comprises two-thirds of the ET; and the isthmus which is the narrowest part of the ET [45]. The isthmus lies between the bony and the fibro-cartilaginous part of the ET. In children, the length of the ET is 1.5 cm and is therefore shorter than in adults. In children, the fibro-cartilaginous part is less than two-third of the total ET length, and the angle of the tube with regards to the skull base is more horizontal (10º) compared to adults (45º) [46].

Under physiological conditions, the bony part, the isthmus, and the pharyngeal orifice of the tube are open for air to pass through. However, the fibro-cartilaginous part is closed under resting conditions. Hence, the ET is normally closed and does not allow air to pass through. The fibro-cartilaginous part of the ET may open during swallowing, yawning or by vocalization due to contraction of the tensor veli palatini muscle [47]. When the ET is open, it allows the middle ear to be connected with the atmospheric space within the nasopharynx, and thus the middle-ear pressure can be regulated. It has been suggested that there is a one-way valve system in the ET, which allows air to flow more readily from the middle-ear to the nasopharynx. This characteristic of the ET seems to be an evolutional defensive mechanism, as potential pathogens from the nasopharynx are prevented to invade the middle-ear. Dysfunction of the ET leads to negative pressure in the middle-ear cavity, which can result into retraction of the tympanic membrane [48]. ET dysfunction can be treated with auto-inflation devices (Otovent^®^) [49], nasal decongestants [50], Eustachian tube balloon dilatation [35], intranasal corticosteroids and Eustachian tuboplasty [51].

Breathing maneuvers can be used to ventilate the middle-ear. Such maneuvers can be used under conditions when the atmospheric pressure is higher than the middle-ear pressure. One of these maneuvers is the Valsalva maneuver, which can be used to ventilate the middle-ear cavity with air from the nasopharynx. The Valsalva maneuver can be performed by closing the nose with the fingers and breathing air against a closed glottis. This will result into buildup of positive pressure in the nasopharynx and will force air to move from the nasopharynx into the middle-ear cavity. It should be noted that an infection in the nose or nasopharynx may spread to the middle-ear with this maneuver [52].

### Flying

**Fig. 3:**
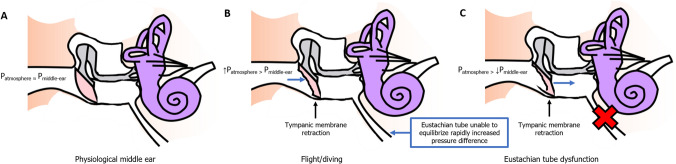
Physiological changes that occur during flight, diving or Eustachian tube dysfunction that cause the tympanic membrane to retract. During flight/diving the atmospheric pressure is higher than the middle ear pressure which causes the middle ear to retract. Eustachian tube dysfunction causes negative pressure in the middle ear which leads to retraction of the tympanic membrane. **a** Normal physiological middle ear, **b** flight/diving, **c** Eustachian tube dysfunction

In flight during the ascend, when the aircraft is reaching high rate of increasing altitude levels (1.5–6.0 km/min), the changes in pressure are well tolerated for pilots and passengers [53]. During ascend, air in the middle-ear expands, generating a relative positive pressure in the middle-ear. The ET allows the middle-ear to ventilate air, causing the middle-ear to equilibrize with atmospheric pressure. However, during descend pilots and passengers may experience discomfort and pain in the ears, along with loss of hearing function. When the aircraft is descending, the atmospheric pressure rapidly increases, while the middle ear is unable to equilibrize the rapid change in pressure between the middle ear cavity and the atmospheric pressure. Air should enter the middle-ear cavity to maintain equilibrium. However, the one-way valve mechanism prevents the middle-ear to be ventilated from the nasopharynx, resulting in a pressure difference between the atmosphere and the middle-ear. This relative difference causes the atmospheric pressure to drive the tympanic membrane inwards to the middle-ear cavity, causing the tympanic membrane to be temporarily retracted [54]. Stangerup et al*.* [55] performed otoscopy on 75 aircraft passengers, of who 10 patients (13%) had a tympanic membrane retraction after flight. To prevent barotrauma, there are several physiological mechanisms (yawning, swallowing, chewing) and maneuvers (e.g., Valsalva) to open the ET, allowing air to be pulled towards the middle-ear. Figure 3 shows the physiological changes that occur during flight and diving which cause the tympanic membrane to retract.

### Diving

A diver is exposed to increased barometric pressures during descend. The barometric pressure at sea level is 100 kPa, which increases linearly by 100 kPa for each 10 m of descent [56]. During descend the barometric pressure outside the middle-ear increases, which must be overcome by equalizing these pressure by opening the ET using equalization maneuvers. Persistent negative pressure in the middle-ear cavity can lead to TMR, extravasation of fluid and hemorrhage in the middle-ear [57]. These fluids in the middle-ear can impair middle-ear ventilation on ascent, which can lead to advancing the barotrauma [11].

Compared to civil aviation, the pressure changes during diving can be more than a few times the atmosphere pressure while during flight this is usually limited to less than one atmospheric pressure. Besides TMRs, divers can also suffer from tympanic membrane perforations and formation of peri-lymphatic fistula, which can lead to hearing loss and vertigo [58]. Peri-lymphatic fistula are caused by rapid pressure changes in endolymphatic pressure or peri-lymphatic pressure. The Valsalva maneuver may increase the intra-labyrinthine fluid pressure via the cochlear aqueduct, but synchronously fail to equilibrate the middle ear pressure [59]. This leads to the intra-labyrinthine fluid pressure to be higher than the middle ear pressure and may cause round window rupture [60].

### Hyperbaric oxygen therapy

During hyperbaric oxygen therapy (HBOT) 100% O_2_ is breath in under pressures that equals 2–3 times the atmospheric pressure and resembles a dive at approximately 15 m underwater [61]. HBOT is frequently used in the military for the treatment of decompression disease and for treatment of acute acoustic trauma [62, 63]. As the physical mechanisms behind HBOT are similar to that of diving, therefore the risk for tympanic membrane retractions and perforations may be similar. Yamamoto et al. [64] described a cohort of 1115 who underwent HBOT, of those patients 165 (14.8%) had otological complications. In total 116 ears were examined by otoscopy, of which 50 ears (4.4%) had a retraction of the tympanic membrane. Inner ear barotrauma was described in one patient (0.1%).

### Limitations

This systematic review has multiple limitations. First, the included studies varied in clinical and methodological characteristics. The included studies were heterogenous in the patient populations, location and severity of the TMR, type of management and type of graft material. Furthermore, the outcomes and TMR classification systems vary among studies which makes it difficult to make any comparison. Second, most studies did not provide adjusted results of potential confounder effects. Therefore, a high risk of confounding might exist in the results. The majority of studies did not use a control group. We identified only two randomized controlled trials, but both were assessed as having a potential high risk of bias. The non-randomized studies were of low evidence quality and the majority of these studies had moderate-to-high risk of bias. This review did not include studies that investigated TMRs with cholesteatoma progression. These patients often receive (radical) mastoidectomy, which has different outcomes for patients with TMRs.

### Clinical relevance for the military patient

In the case of the navy cadet that was mentioned in the background section, we chose to operate the mild TMR. He successfully underwent endoscopic tympanoplasty which was without complications. After 3 years of follow-up, the hearing remained stable and the TMR has been resolved. The outcome for this cadet after the intervention was excellent and the cadet was able to perform its duties. However, the watchful waiting option would also have been justified in this case. This wait-and-see policy would allow the patient to be followed-up and, in case of hearing loss or progression of the TMR, a decision to operate can still be considered. The level of evidence of the studies regarding the management of TMRs is relatively low. Based on the Consensus Based Practice Guide by Yung and Neumann, it is thought (84.6% agreement percentage) that in an asymptomatic mild retraction (Sade grade I) surgical treatment might not be superior to the wait-and-see policy [65]. In recent years, four studies [5, 31, 66] were published that used the wait-and-see policy for the management of TMRs. It was found that the majority of TMRs (> 75%) remain stable in terms of otoscopy and audiology. Patients presenting with a mild TMR (Sade grade I) seem to recover to acceptable hearing levels. Whereas the excision of the TMR with ventilation tube insertion, creates persistent perforations in 3–13% and TMR recurrences in 11–25% of operated patients. This suggest that indeed in mild retractions (Sade grade I) a surgical management strategy might not be superior to the wait-and-see policy. For mild and asymptomatic retractions, the wait-and-see policy might be the favorable management option. A prospective comparative study is needed to confirm this. For symptomatic and more severe TMRs (Sade grade III-IV), it is unclear whether the wait-and-see policy is superior to surgery. For these patients, a surgical intervention might be necessary to reduce hearing loss and reduce the risk of cholesteatoma development. It is not clear which surgical management strategy is the most ideal treatment option for these patients. The available management options should be discussed with the patient in shared decision-making. Several factors such as the age of the patient, amount of hearing loss, risk of cholesteatoma formation and severity and location of the TMR should be considered. For military patients, additional factors should be considered such as the specific duty requirements of the soldier and exposure to high pressure changes (e.g., air force pilots and navy divers).

## Conclusion

This study provides an overview of all the studies that have been published on the surgical management and wait-and-policy for tympanic membrane retractions. The evidence for the management of tympanic membrane retractions is heterogenous and depends on many factors such as the patient population, location, and severity of the TMR and presence of other ear pathologies (e.g., perforation, cholesteatoma and serous otitis media). For mild retractions, interventions might not outweigh the wait-and-see policy in terms of clinical outcomes. Prospective studies are needed to confirm this.

### Search strategy

#### PubMed

((tympanic [tiab] OR retrotympanic [tiab] OR epitympanic [tiab]) AND membrane [tiab]) OR (tympanic membrane) OR (eardrum* [tiab] OR (ear* [tiab] AND drum* [tiab])) AND ((retract* [tiab] OR collaps* [tiab]OR atelectas* [tiab] OR atelectat*[tiab]) OR retraction pocket) AND ("Surgical Procedures, Operative"[Mesh] OR (surg* [tiab] OR excis* [tiab] OR reconstruct* [tiab]) OR (ventilation [tiab] OR grommet*[tiab] OR natural course OR watchful OR wait-and-see OR mastoidectom* [tiab] OR tympanoplast* [tiab] OR myringotom* [tiab] OR tube* [tiab] OR tympanostom* [tiab])).

#### Embase

('eardrum' OR (eardrum* OR (ear* AND drum*)) OR ((tympanic OR retrotympanic OR epitympanic) AND membrane) OR (pars AND (tensa OR flaccida))) AND (retract* OR collaps* OR atelectas* OR atelectat*) AND (surg* OR excis* OR reconstruct* OR ventilation OR grommet* OR mastoidectom* OR tympanoplast* OR myringotom* OR tube* OR tympanostom*).

## Supplementary Information

Below is the link to the electronic supplementary material.

Supplementary file1 (DOCX 14 KB)

Supplementary file2 (DOCX 14 KB)

Supplementary file3 (DOCX 239 KB)

Supplementary file4 (DOCX 604 KB)

Supplementary file5 (DOCX 14 KB)

Supplementary file6 (DOCX 18 KB)

Supplementary file7 (DOCX 15 KB)

Supplementary file8 (DOCX 19 KB)
