# Supplementary material for: Management of tympanic membrane retractions: a systematic review
Source: Eur Arch Otorhinolaryngol. 2021 Mar 10;279(2):723–37. doi: 10.1007/s00405-021-06719-3 (PMC8794915; doi:10.1007/s00405-021-06719-3)
Supplement: Supplementary file 1 — Supplementary file1 (DOCX 14 KB) [file 405_2021_6719_MOESM1_ESM.docx]

**Supplement 2.** Risk of bias assessment of Non-Randomized studies.^11^

| **Stage of bias** | **Category of bias** | **Bias domain** |
| --- | --- | --- |
| ***Preintervention*** | Confounding | Bias due to confounding |
| ***Preintervention*** | Selection bias | Bias in selection of participants into the study |
| ***At intervention*** | Information bias | Bias in classification of interventions |
| ***Postintervention*** | Confounding | Bias due to deviations from intended interventions |
| ***Postintervention*** | Selection bias | Bias due to missing data |
| ***Postintervention*** | Information bias | Bias in measurement of outcomes |
| ***Postintervention*** | Reporting bias | Bias in selection of the reported result |
| ***Overall risk of bias*** | All types of bias | Overall bias |
