# Supplementary figures and images for: Management of tympanic membrane retractions: a systematic review
Source: Eur Arch Otorhinolaryngol. 2021 Mar 10;279(2):723–37. doi: 10.1007/s00405-021-06719-3 (PMC8794915; doi:10.1007/s00405-021-06719-3)

**Supplement 3.** Weighted summary plot of non-randomized studies.


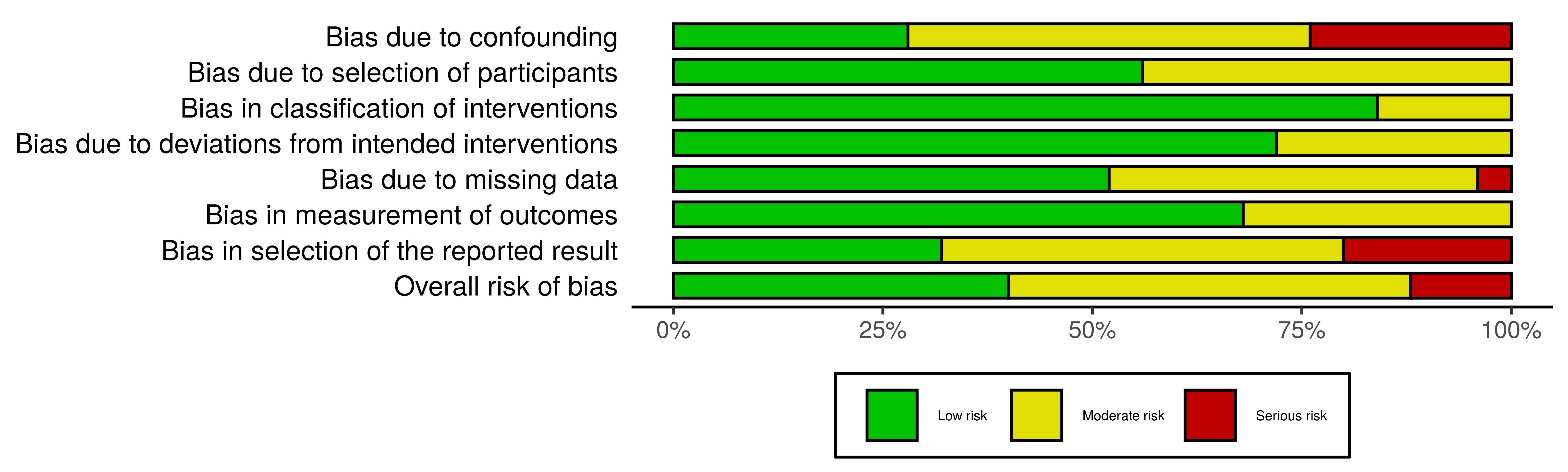

Supplement: Supplementary file 2 — Supplementary file2 (DOCX 14 KB) [file 405_2021_6719_MOESM2_ESM.docx]

**Supplement 4.**Traffic light plot of risk of bias of the randomized controlled trials

(n = 2, 76 patients).

***
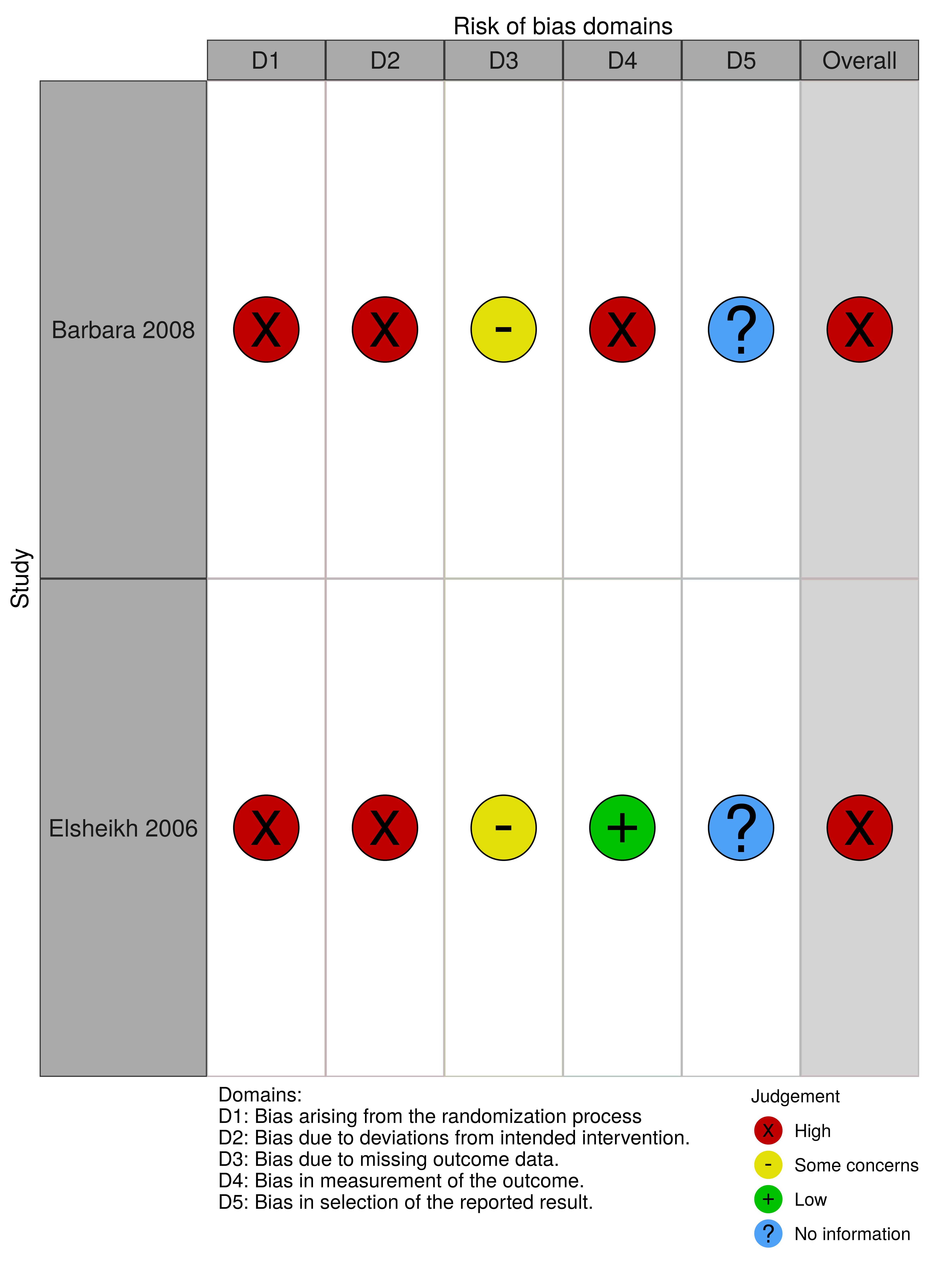
***

Supplement: Supplementary file 3 — Supplementary file3 (DOCX 239 KB) [file 405_2021_6719_MOESM3_ESM.docx]
