# Supplementary material for: Management of tympanic membrane retractions: a systematic review
Source: Eur Arch Otorhinolaryngol. 2021 Mar 10;279(2):723–37. doi: 10.1007/s00405-021-06719-3 (PMC8794915; doi:10.1007/s00405-021-06719-3)
Supplement: Supplementary file 4 — Supplementary file4 (DOCX 604 KB) [file 405_2021_6719_MOESM4_ESM.docx]

***Supplement 5.*** Risk of bias assessment of Randomized Controlled Trials

(n = 2, 76 patients).

| **Study** | **Domain** | **Support for judgment & review authors’ judgment** |
| --- | --- | --- |
| **Barbara *et al.* (2008)** | Random sequence generation | High, Sequential allocation on a consecutive cohort. |
|  | Allocation concealment | High, Sequential allocation to each of the treatment arms would allow the investigator to know which arm the subsequent patient would be allocated to. |
|  | Blinding of participants and personnel | Some concerns, the study is performed by a single investigator. No blinding took place. |
|  | Blinding of outcome assessment | High, five patients from the control group were not included in the analysis as they were lost to follow up. |
|  | Incomplete outcome data | High, five patients from the control group were not included in the analysis as they were lost to follow up. |
|  | Selective reporting | Unclear, all outcome measures only assessed by the investigator (i.e. otoscopic progression and hearing assessment). |
|  | Other sources of bias | Unclear, single investigator who performed the randomization, treatment and assessment at follow up. |
| **Elsheikh *et al*. (2006)** | Random sequence generation | High, no description of randomization protocol. |
|  | Allocation concealment | High, no evidence of allocation concealment. |
|  | Blinding of participants and personnel | High, no comment that the authors were blinded to patient allocation. |
|  | Blinding of outcome assessment | High, no comment that the authors were blinded to outcome allocation. |
|  | Incomplete outcome data | Low, no patients lost to follow up. |
|  | Selective reporting | Unclear risk, unclear who performed the postoperative otoscopic assessment or audiological testing. No grading given for postoperative results. |
|  | Other sources of bias | Low |
