# Supplementary material for: Management of tympanic membrane retractions: a systematic review
Source: Eur Arch Otorhinolaryngol. 2021 Mar 10;279(2):723–37. doi: 10.1007/s00405-021-06719-3 (PMC8794915; doi:10.1007/s00405-021-06719-3)
Supplement: Supplementary file 5 — Supplementary file5 (DOCX 14 KB) [file 405_2021_6719_MOESM5_ESM.docx]

**Supplement 6. Study characteristics of included studies.**

| **Author (year)** | **Study design** | **Participants** | **Intervention** | **Outcomes** |
| --- | --- | --- | --- | --- |
| **Barbara (2008)** | RCT | Setting: university hospital  Age: Adults  Country: Italy | The surgical procedure consisted of a retroauricular approach, removal of a small piece of tragal cartilage, cleansing of the epitympanic pocket until denudation of the ossicular components, and placement of the cartilage graft to reconstruct the lateral epitympanic wall. | Otoscopy  Audiometry  Tympanometry |
| **Elsheikh (2006)** | RCT | Setting: university hospital  Age: Children and Adults  Country: Egypt | In Group I, 23 patients underwent reconstruction of the TM with perichondrium/cartilage graft and intraoperative T-tube insertion and in Group II, 23 patients underwent reconstruction of the TM with perichondrium/cartilage graft without ventilation tube insertion. | Graft success,  Audiometry  Postoperative complications |
| **Parab (2019)** | PS | Setting: university hospital  Age: Children and Adults  Country: India | Endoscopic Tympanoplasty | Audiometry |
| **Kalra (2018)** | PS | Setting: regional hospital  Age: Children and Adults  Country: India | Excision and cartilage tympanoplasty | Audiometry  Otoscopy  Recurrence of perforation |
| **Bayoumy (2020)** | RS | Setting: Regional hospital  Age: Children and Adults  Country: The Netherlands | Wait-and-see policy | Audiometry  Otoscopy  Cholesteatoma rate  Perforation rate |
| **Parkes (2018)** | RS | Setting: university hospital  Age: Children with cleft palate  Country: US | Wait-and-see policy | Audiometry  Otoscopy  Tympanometry |
| **Cutajar (2018)** | RS | Setting: university hospital  Age: Children and Adults  Country: UK | Wait-and-see policy | Audiometry  Otoscopy  Cholesteatoma rate |
| **Comacchio (2017)** | RS | Setting: university hospital  Age: Children and Adults  Country: Italy | Tympanoplasty with perichondral cartilage graft. | Otoscopy  Cholesteatoma rate  Ossicular erosion  Recurrence rate |
| **Noij (2017)** | PS | Setting: regional hospital  Age: Adults  Country: | The majority of middle ear operations were performed transmeatally, and two retraction pockets required a combined mastoidectomy approach for a cholesteatoma was present that could not be resected completely via the transmeatal approach. | Audiometry  Otoscopy |
| **Kasbekar (2014)** | RS | Setting: regional hospital  Age: Children and Adults  Country: UK | Surgical Technique: Modified Cartilage Tympanoplasty | Audiometry |
| **Rath (2011)** | PS | Setting: university hospital  Age: Children  Country: Hungary | Transmeatal excision of pars tensa retraction pockets with simultaneous ventilation tube insertion. | Audiometry  Otoscopy |
| **Cassano (2010)** | RS | Setting: university hospital  Age: Children  Country: Italy | Grade I and II retractions were treated with medical therapy or ventilation tube insertion; in III or IV grade retractions, excision and tympanic reinforcement with cartilage grafting and in some cases ossiculoplasty were performed. | Audiometry  Otoscopy |
| **Özbek (2010)** | RS | Setting: regional hospital  Age: Children  Country: Turkey | Type1 cartilage tympanoplasty performed with the palisade technique compared with primary Type 1 tympanoplasty performed with temporalis fascia. | Audiometry  Otoscopy  Perforation rate |
| **Borgstein (2009)** | RS | Setting: university hospital  Age: Children  Country: The Netherlands | Tympanoplasty with transmeatal approach and tragal perichondrium, or temporalis fascia grafting. | Audiometry |
| **Borgstein (2008)** | RS | Setting: university hospital  Age: Children  Country: The Netherlands | Excision of tympanic membrane retraction with transmeatal approach and grafting with tragal perichondrium. | Audiometry |
| **Borgstein (2008)** | RS | Setting: university hospital  Age: Children  Country: The Netherlands | Excision of tympanic membrane retraction without grafting. | Audiometry  Otoscopy |
| **Dornhoffer (2003)** | RS | Setting: university hospital  Age: Children  Country: US | Cartilage reconstruction of the TM with the perichondrium cartilage island flap or the palisade technique. | Audiometry |
| **Couloginer (2003)** | RS | Setting: university hospital  Age: Children  Country: Belgium | Excision of the pocket and tympanic reinforcement with a tragal or conchal cartilaginous graft | Audiometry |
| **Dornhoffer (2000)** | RS | Setting: university hospital  Age: Adults and Children  Country: US | Perichondrium cartilage tympanoplasty.  Type I tympanoplasty  Tympanoplasty with reconstruction using partial ossicular replacement prosthesis.  Tympanoplasty with reconstruction using total ossicular replacement prosthesis. | Audiometry |
| **Srinivasan (2000)** | RS | Setting: regional hospital  Age: Children  Country: UK | Excision of tympanic membrane and ventilation tube insertion. | Audiometry  Recurrence rate  Perforation rate |
| **Blaney (1999)** | RS | Setting: university hospital  Age: Children  Country: UK | Excision of tympanic membrane and ventilation tube insertion. | Audiometry  Otoscopy |
| **Yung (1997)** | RS | Setting: regional hospital  Age: Adults  Country: United Kingdom | Cartilage-perichondral tympanoplasty  Type III tympanoplasty  Type IV tympanoplasty | Recurrence free rate  Cholesteatoma rate  Audiometry |
| **Harner (1995)** | RS | Setting: university hospital  Age: Children and Adults  Country: US | Tragal cartilage tympanoplasty | Audiometry |
| **Walsh (1995)** | RS | Setting: regional hospital  Age: Children  Country: UK | Excision of tympanic membrane and ventilation tube insertion. | Audiometry  Otoscopy |
| **Mills (1991)** | PS | Setting: regional hospital  Age: Children and Adults  Country: UK | Tympanoplasty with cartilage/perichondrium grafts. | Audiometry  Otoscopy |
| **Luntz (1991)** | RS | Setting: university hospital  Age: Children and Adults  Country: Israel | Tympanoplasty operation with or without ossiculoplasty | Otoscopy |
| **Avraham (1991)** | RS | Setting: university hospital  Age: Children and Adults  Country: Israel | Tympanoplasty with/without masteoidectomy | Otoscopy |
