# Supplementary material for: Management of tympanic membrane retractions: a systematic review
Source: Eur Arch Otorhinolaryngol. 2021 Mar 10;279(2):723–37. doi: 10.1007/s00405-021-06719-3 (PMC8794915; doi:10.1007/s00405-021-06719-3)
Supplement: Supplementary file 6 — Supplementary file6 (DOCX 18 KB) [file 405_2021_6719_MOESM6_ESM.docx]

**Supplement 7. Study characteristics of excluded studies.**

| **Author** | **Year** | **Reason for exclusion** |
| --- | --- | --- |
| ***Anderson*** | ***2004*** | **Cholesteatoma surgery** |
| ***Karchier*** | ***2016*** | **Cholesteatoma surgery** |
| ***Rosito*** | ***2016*** | **Cholesteatoma surgery** |
| ***Sakagami*** | ***1994*** | **Cholesteatoma surgery** |
| ***Sade*** | ***1988*** | **Cholesteatoma surgery** |
| ***Sade*** | ***1993*** | **Cholesteatoma surgery** |
| ***Prescott*** | ***1999*** | **Cholesteatoma surgery** |
| ***Sadé*** | ***2002*** | **Cholesteatoma surgery** |
| ***Rosito*** | ***2017*** | **Cholesteatoma surgery** |
| ***Neff*** | ***2014*** | **Cholesteatoma surgery** |
| ***Pauna*** | ***2014*** | **Cholesteatoma surgery** |
| ***Jesic*** | ***2014*** | **Cholesteatoma surgery** |
| ***Abdelghany*** | ***2013*** | **Perforation surgery** |
| ***Albirmawy*** | ***2010*** | **Perforation surgery** |
| ***Bernal-Sprekelsen*** | ***2003*** | **Perforation surgery** |
| ***Bernardeschi*** | ***2017*** | **Perforation surgery** |
| ***D'Eredità*** | ***2009*** | **Perforation surgery** |
| ***Redaelli de Zinis*** | ***2015*** | **Perforation surgery** |
| ***Acuin*** | ***2015*** | **Perforation surgery** |
| ***Gupta*** | ***2015*** | **Perforation surgery** |
| ***Lou*** | ***2020*** | **Perforation surgery** |
| ***Abdullah*** | ***2007*** | **Otitis media** |
| ***Beutner*** | ***2010*** | **Otitis media** |
| ***Bluestone*** | ***1984*** | **Otitis media** |
| ***Charachon*** | ***1992*** | **Otitis media** |
| ***Diacova*** | ***2007*** | **Otitis media** |
| ***Hol*** | ***2010*** | **Otitis media** |
| ***Hartzell*** | ***2010*** | **Otitis media** |
| ***Vlastarakos*** | ***2007*** | **Otitis media** |
| ***Caye-Thomasen*** | ***2008*** | **Otitis media** |
| ***Garov*** | ***2016*** | **Otitis media** |
| ***Grewal*** | ***2003*** | **Otitis media** |
| ***Karma*** | ***1982*** | **Otitis media** |
| ***Koch*** | ***1981*** | **Other therapies** |
| ***Daudia*** | ***2010*** | **Other therapies** |
| ***Pestalozza*** | ***1974*** | **Other therapies** |
| ***Nankivell*** | ***2010*** | **Review** |
| ***Yung*** | ***2011*** | **Review** |
