# Supplementary material for: Management of tympanic membrane retractions: a systematic review
Source: Eur Arch Otorhinolaryngol. 2021 Mar 10;279(2):723–37. doi: 10.1007/s00405-021-06719-3 (PMC8794915; doi:10.1007/s00405-021-06719-3)
Supplement: Supplementary file 7 — Supplementary file7 (DOCX 15 KB) [file 405_2021_6719_MOESM7_ESM.docx]

**Supplement 1.** Risk of bias assessment of Randomized Controlled Trials.^39^

| **Bias domain** | ***Source of judgment*** | ***Support for judgment*** |
| --- | --- | --- |
| ***Selection bias*** | Random sequence generation  Allocation concealment | Describe the method used to generate the allocation sequence in sufficient detail to allow an assessment of whether it should produce comparable groups.  Describe the method used to conceal the allocation sequence in sufficient detail to determine whether intervention allocations could have been foreseen before or during enrolment. |
| ***Performance bias*** | Blinding of participants and personnel | Describe all measures used, if any, to blind trial participants and researchers from knowledge of which intervention a participant received. Provide any information relating to whether the intended blinding was effective. |
| ***Detection bias*** | Blinding of outcome assessment | Describe all measures used, if any, to blind outcome assessment from knowledge of which intervention a participant received. Provide any information relating to whether the intended blinding was effective. |
| ***Attrition bias*** | Incomplete outcome data | Describe the completeness of outcome data for each main outcome, including attrition and exclusions from the analysis. State whether attrition and exclusions were reported, the numbers in each intervention group (compared with total randomized participants), reasons for attrition or exclusions where reported, and any re-inclusions in analyses for the review. |
| ***Reporting bias*** | Selective reporting | State how selective outcome reporting was examined and what was found. |
| ***Other bias*** | Other biases | State any important concerns about bias not covered in the other Bias due to problems not covered elsewhere domains in the tool. |
